# Heterotopic ossification after hip arthroscopy

**DOI:** 10.1093/jhps/hnv052

**Published:** 2015-08-28

**Authors:** Eyal Amar, Zachary T. Sharfman, Ehud Rath

**Affiliations:** Division of Orthopedic Surgery at Tel-Aviv Sourasky Medical Center and the Sackler Faculty of Medicine, Tel Aviv University, Tel Aviv, Israel

## Abstract

Heterotopic ossification (HO) after hip arthroscopy is the abnormal formation of mature lamellar bone within extra skeletal soft tissues. HO may lead to pain, impaired range of motion and possibly revision surgery. There has been a substantial amount of recent research on the pathophysiology, prophylaxis and treatment of HO associated with open and arthroscopic hip surgery. This article reviews the literature on the aforementioned topics with a focus on their application in hip arthroscopy.

## INTRODUCTION

Heterotopic ossification (HO) is the abnormal formation of mature lamellar bone within extraskeletal soft tissues. HO after hip arthroscopy may impede functional outcomes by causing pain, impingement and decreased range of motion. HO is one of the most common complications after hip arthroscopy with reported rates of 0–44% without prophylaxis [1–5]. Lesions range from small clinically insignificant foci of ossification to large deposits of bone that can cause stiffness and discomfort, ultimately compromising surgical outcomes. Revision surgery to excise HO may be required in patients who are experiencing refractory pain and/or restricted motion [4]. Despite the high incidence of HO and multiple clinical studies examining the phenomenon, the pathophysiology and etiology of HO remain unclear.

## PATHOGENESIS AND MECHANISM OF HO

The pathological mechanism responsible for HO has been widely studied, yet a definitive mechanism has not been established. Current and historical research has illuminated the likely cellular linage responsible for HO and the cellular mechanism that most likely contributes to this pathologic ectopic tissue.

### Candidate cells responsible for HO

Few studies evidence an ectodermal or endodermal cell origin for the mechanism and pathophysiology of HO [6–9]. Cells of ectodermal and endodermal origin may indirectly contribute to HO but it is unlikely that they directly give rise to heterotopic osteolineage cells [10]. Although endodermal cells may not be directly responsible for HO [11, 12], endodermal cells have been shown to affect HO through paracrine secretions and other cellular crosstalk mechanisms [13–18].

The most likely candidate cell responsible for HO is the mesenchymal stem cell (MSC) [10, 12, 19]. Wosczyna *et al*. [12], found that these cells consistently incorporated into areas of osteogenic and chondrogenic foci. Kan *et al*. [10] recently establish that the cells partaking in physiologic bone formation may not be implicated in the mechanism of HO.

### Proposed mechanism

**Fig. 1.**
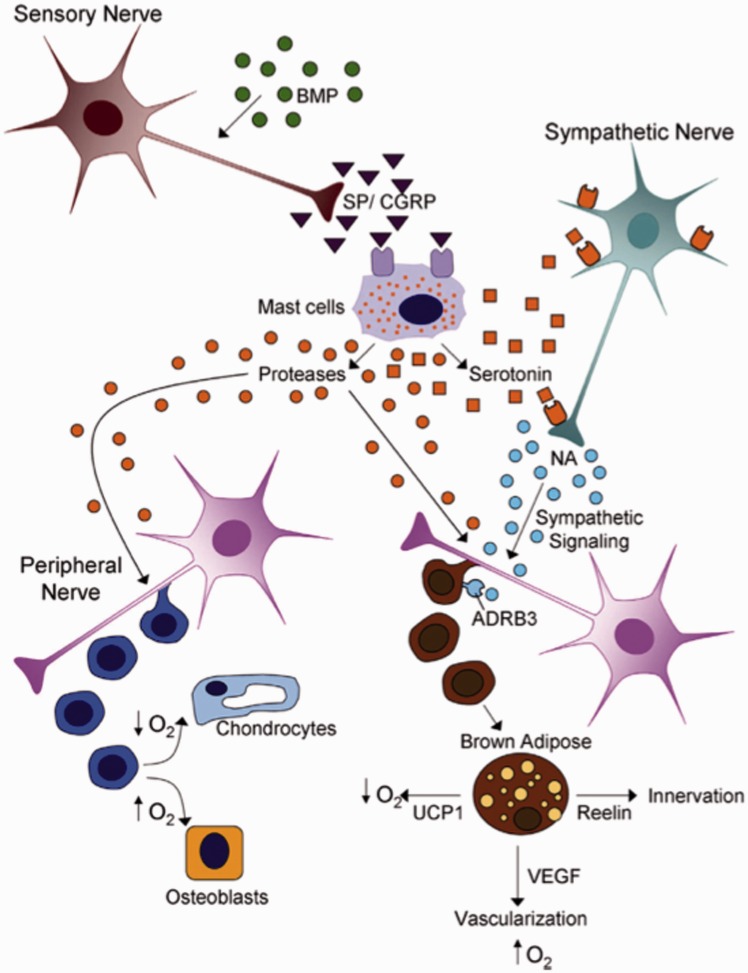
Heterotopic ossification is initiated local tissue damage leading to increased BMP-2 targeting of sensory nerves. Subsequently, a cascade of molecular mechanisms including the binding of SP and CGRP to mast cell and sympathetic signaling induces the remodeling of peripheral nerves. This remodeling initiates the production of chondro-osseous, glial, vascular and neural progenitor cells. These new cells respond to signals from transient brown adipocytes that regulate local oxygen content, vascularization and innervation to produce HO. (Reproduced with permission from reference [9].)

The definitive mechanism responsible for HO has not yet been established. However, inflammatory and traumatic processes have been implicated and scientifically substantiated as contributors to HO. The release of bone morphogenic protein 2 (BMP2) has been well established as an instigator of HO [19, 20] BMP2 is most likely released upon injury [21–23]and mediates an increase in inflammatory markers Substance P (SP) and calcitonin gene-related protein (CGRP) resulting in the recruitment of immune cells such as neutrophils, mast cells and platelets [20]. The resultant degranulation of mast cells, the increased activity of proteases and the presence of activated metaloproteases provoke local tissue disturbance. BPM2 has further been implicated to initiate the molecular pathway responsible for differentiation of peripheral nerve perineurium progenitor cells to brown adipose like cells, essential for nerve remodeling and vascularization vital to the formation of HO [20]. After perineurium type cells migrate to the site of inflammation and differentiate to brown adipose tissue type cells the increased oxygen requirements of the brown adipose tissue type cells establishes a hypoxic microenvironment favorable for chondrogenesis [24]. The microenvironment necessary for osteogenesis; however, requires a less hypoxic environment. Brown adipose type cells were also shown to express vascular endothelial growth factors, which contribute to new vasculature, oxygenating the hypoxic microenvironment and allowing for osteogenesis [25]. Figure 1 represents the molecular mechanism for HO.

### Risk factors

Although risk factors predisposing the development of HO have been identified, only a handful of those identified have been validated in large studies. The majority of those risk factors evaluated where in patients after total hip arthroplasty (THA). The risk factors for HO in THA include patient related factors such as male gender [26], previous hip surgery [27] and history of HO [28]. Bone producing diseases such as ankylosing spondylitis [29], diffuse idiopathic skeletal hyperostosis and hypertrophic osteoarthritis [30] were also identified as risk factors for HO after THA. Additional considerations such as the surgical approach are important to HO formation, as it was shown that the lateral or anterolaeral approach carries greater risk for HO development than the posterior approach [31, 32]. The use of trochanteric or femoral osteotomy has also been shown to increase the risk for HO [33, 34]. Other non-arthroplasty related risk factors include brain injuries (e.g. head trauma, cerebrovascular accident), spinal cord or lower motor neuron injuries, soft tissue injuries (e.g. blunt trauma, joint dislocation), vascular diseases (e.g. atherosclerosis, valvular heart disease) and arthropathies [35].

Studies concerning the risk factor for HO formation in hip arthroscopy are scarce. To date, there are only two studies that examined factors (excluding pharmacologic prophylaxis) that may affect HO incidence after hip arthroscopy [36, 37]. Randelli *et al*. [3] suggested capsular incision to be a relevant predisposing factor. Amar *et al*. [37, p. 1124] showed that capsular repair failed to decrease the rate of HO after hip arthroscopy. Randelli *et al*. [3] noted that extensive rim trimming, anchor placement, and male gender to be the also relevant predisposing factors, and Bedi *et al*. [4, p. 685] noted that HO was more prevalent in male patients who underwent osteochondroplasty. Beckman *et al*. [36] retrospectively reviewed 357 consecutive cases of hip arthroscopy over a 3-year period. The authors identified both mixed-type femoroacetabular impingement (FAI) resection and the absence of non-steroidal anti-inflammatory drug (NSAID) prophylaxis as important predictors of HO development.

### Classifications

The Brooker classification based on anteroposterior radiographs is commonly used to determine the level of HO [38]. Grade 1 represents small islands of bone within the soft tissues, grade 2 represents bone islands between the pelvis and femur with >1 cm between the bone surfaces, grade 3 represents bone islands that reduce the space between the pelvis and femur to <1 cm and grade 4 represents complete ankylosis of the hip. This classification has been criticized because bone that appears to be bridging may actually be located either anterior or posterior to the hip and thus does not cause significant reduction to the range of motion.

### Clinical manifestations

The presence of small to medium size foci of HO is generally asymptomatic. However, large foci of HO may cause functional impairment in some patients. Beckman *et al*. [36] considered HO to be symptomatic for 9 of the 34 cases identified in his study. Bedi *et al*. [4] reported 7 cases of ectopic bone removal among 29 hips that developed HO at a mean duration of 11.6 months after hip arthroscopy.

The clinical manifestations of HO after hip arthroscopy are difficult to isolate from other sources of post-operative pain. Stiffness may result as part of scar formation with or without ectopic bone formation. A mechanical blockage can explain limited range of motion if the ectopic bone has been formed in the plane of motion—mainly anterior and lateral. Larson [1] reported one case of significant motion limitation resulting from ossification of the iliopsoas tendon. This motion deficit was nearly completely resolved at 1 year, and no further treatment was required at the most recent follow-up. The gain in range of motion after hip replacement surgery was significantly less in patients with class III or IV heterotopic bone formation than in those without heterotopic bone formation. However, heterotopic bone formation had no serious impact on hip muscle strength [26]. No difference with regard to functional outcomes after hip arthroscopy was found between those patients who developed HO and those who did not [5].

### Imaging and laboratory findings

**Fig. 2.**
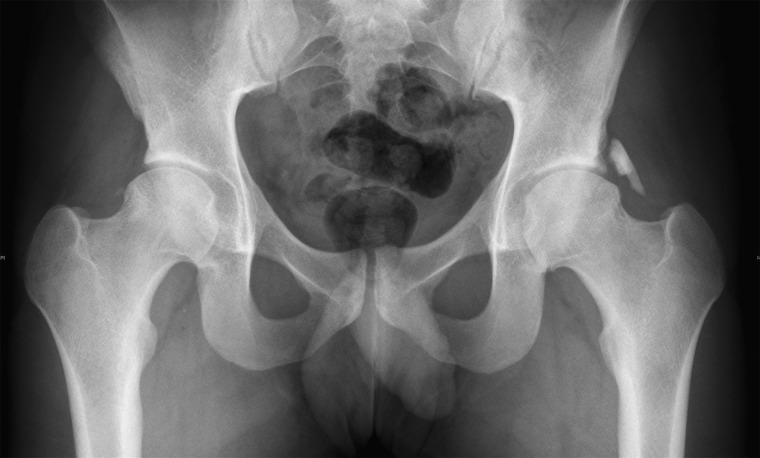
Follow up radiograph of a 20-year-old patient after bilateral hip arthroscopy. The radiograph was taken 9 months status post left and 3 months status post right hip arthroscopy. HO on the left side was evident on radiographs 10 weeks after the index procedure. NSAID prophylaxis using etodolac 600 mg once daily for 2 weeks was administered only after the operation on the right hip.

**Fig. 3.**
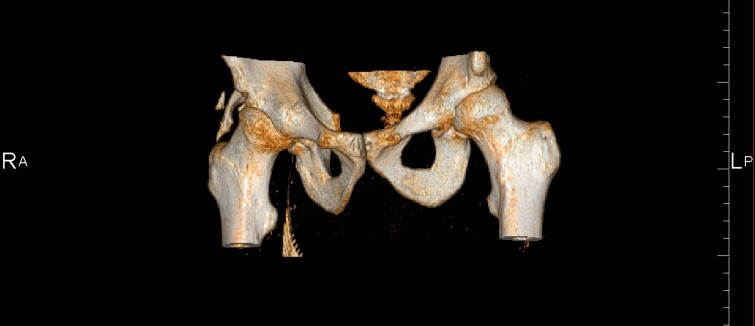
3D CT reconstruction of a 34-year-old triathlete showing grade 3 HO with acetabular origin and posterolateral location of the HO.

Imaging plays an important role in diagnosis and optimizing the timing of HO excision surgery. Plain radiographs and computed tomography (CT) imaging are the most reliable methods to evaluate advanced ossification foci. HO can present on post-operative radiographs as early as 2 weeks post-surgery. However, in a study looking at the incidence of HO after hip arthroscopy patients diagnosed with HO via X-ray at 9 weeks after surgery did not have radiographic evidence of HO on the 2-week post-operative radiograph [5]. Ossification observed by radiography typically appears as a cloud like hyper-density and gradually matures to solid bone by 3 months (Fig. 2). CT may show a soft tissue mass, followed by visualization of bone earlier than can be seen with conventional radiographs. During surgical planning, assessing the severity of ossification is crucial, as resection must be performed only when HO is mature. Although both plain radiographs and CT imaging are the standard references used to assess HO maturity, CT and 3D CT more accurately define the different stages of ossification than plain radiographs. In the case of revision surgery for the removal of HO, CT is essential for surgical planning and visualizing the shape and spatial location of the ectopic bone [39] (Fig. 3). Magnetic resonance imaging is typically not used in the diagnosis and assessment of HO. The signal intensity characteristics of pelvic HO change in various stages of HO maturation. With progressive maturity of HO, T2 signal intensity and contrast enhancement decrease but fat and cortical bone-equivalent signal intensity increase [40].

Ultrasonography is not generally employed to assess HO once the diagnosis is established. Ultrasound typically shows abnormalities in muscle as a chaotic disruption of the normal lamellar structure. Evidence of HO can be seen up to 10–14 days before radiographic evidence of HO appears [41]. Assessing HO using three-phase bone scintigraphy with technetium-99 m methylene diphosphonate may detect HO 2–6 weeks earlier than detection with standard X-ray radiography. Early in the course of HO, only the blood pool images may be positive whereas abnormal uptake during the soft tissue phase is diagnostic later in the course of the disease. Activity on delayed bone scans usually peaks a few months after injury, after which the intensity of activity on these scans progressively lessens, with a return toward normal at 6–12 months [42].

In the case of HO after trauma, blood alkaline phosphatase levels become abnormal ∼2 weeks post-injury and may reach 3.5 times the normal value 10-week after injury. These levels may return to normal values at ∼18 weeks after the injury [42].

### Prophylaxis

Once an HO lesion is present, the continued formation and maturation of the lesion cannot be prevented by non-surgical measures. Consequently, either external beam radiation (EBR) or pharmacologic agent prophylaxis has gained acceptance to combat the initial formation of HO. Ionizing radiation exerts its influence on rapidly dividing cells by altering the structure of nuclear DNA. Thus, early post-operative radiation may prevent differentiation of the pluripotent mesenchymal cells into pathologic osteoblasts [43]. Both post- and pre-operative EBR are clinically effective in reducing the incidence of HO following hip surgery [43–46]. Commonly used protocols of EBR include post-operative, single-dose regimens of 600–800 Gy performed by post-operative day 3 as well as pre-operative single dose regimens of 800 Gy performed within 6 h prior to surgery [47]. Currently, there are no studies evaluating the efficacy of prophylactic irradiation for HO after hip arthroscopy.

Pharmacologic agents proposed for HO prophylaxis consists of non-selective cyclooxygenase (COX) inhibitors, selective COX-2 inhibitors, aspirin, BMP type 1 receptor inhibition and BMP antagonist, nuclear retinoic acid receptor γ agonists (RAR-γ), free radical (FR) scavengers and bisphosphonates. Excluding NSAIDs the abovementioned agents are not regularly used for HO prophylaxis regardless the index operation.

The prevention of HO using aspirin has demonstrated mixed results. One prospective study of 2649 patients deemed aspirin ineffective in HO prevention [48] while two other retrospective studies found aspirin to be superior to Coumadin in HO prevention after THA [49, 50]. BMP1 receptor inhibitors, BMP antagonist, RAR-γ agonists and FR scavengers have yet to be substantiated as efficacious for HO prophylaxis in human studies. Therefore, those measures currently offer no clinical use as post-operative HO prophylactic measures [51].

Biphosphonate (i.e. etidronate disodium and ethylhydroxydiphosphonate) treatment resulted in delay rather than prevention of osteoid mineralization and its use as a prophylactic measure was thus discontinued due to ineffectiveness [52, 53].

NSAIDs inhibit the conversion of arachidonic acid to prostaglandins by COX enzymes and thereby inhibit prostaglandin production. COX enzymes are found in two isoforms with differing distribution and expression. The COX-2 isoform, which is more frequently expressed in pro-inflammatory states, can be selectively targeted. Specifically targeting this isoform in prophylactic treatment of inflammatory conditions may be advantageous as it avoids many of the adverse side effects attributed to the simultaneous COX-1 and COX-2 inhibition by non-selective NSAIDs [54].

The rate of HO after hip arthroscopy without prophylaxis may be as high as 44% [5]. To date, only a limited number of studies have evaluated the role of NSAIDs (selective and non-selective) in HO prevention after hip arthroscopy [3, 4, 36]. Furthermore, the optimal NSAID agent as well as the optimal duration of treatment has yet to be determined. Randelli *et al*. [3] evaluated the efficacy of various NSAID agents as prophylaxis in patients post-hip arthroscopy. Five hips presented with HO, with overall prevalence of 1.6%. All five patients with HO belonged to the control group. The authors therefore concluded that NSAID prophylaxis for HO after arthroscopic FAI treatment proved to be an effective preventative measure (Table I).

**Table I.** A literature review of relevant articles regarding the rates of HO after hip arthroscopy with and without prophylactic measures

Author	Prophylaxis	HO (%)	Male/Female	Excision surgery
Larson *et al*. [1]	None	6/96 (6.2%)	54/42	None of the patients required surgical excision.
Clohisy *et al*. [2]	None	4/35(11.4%)	28/7	None of the patients required surgical excision
Randelli *et al*. [3]	15 patients—etoricoxib 90 mg × 1/day for 3 weeks 248 patients—naproxen 500 mg × 2/day for 3 weeks 22 patients—other NSAIDs (aceclofenac, ketoprofen, indomethacine) for 3 weeks 15 patients—no prophylaxis (control)	Study—0/285 (0%) Control—5/15 (33%)	Control—9/6 Study—171/114 HO—4/1	NA
Beddi *et al*. [4]	Protocol 1—277 patients—naproxen 500 mg × 2/day for 30 days Protocol 2—339 patients—Indomethacine 75 mg daily for 4 days + Protocol 1	Protocol 1—23/277(8.3%) Protocol 2—6/339(1.8%)	Protocol 1—154/123 Protocol 2—188/151 HO—21/8	7 patients 6 received protocol 1 Mean time to surgery—11.6 months Male/Female—6/1
Rath *et al*. [5]	None	22/50(44%)	31/19 HO—15/7	None of the patients required surgical excision
Beckman *et al*. [36]	Control—92 patients 196 patients—naproxen 500 mg × 2/day for 3 weeks	Control—23/92(25%) Study—11/196(5.6%)	Control—41/51 Study—75/121	9 patients Control—4 patients Study—5 patients Mean time to surgery > 12 min

Bedi *et al.* [4] compared two HO prophylaxis protocols for hip arthroscopy patients. The majority of HO cases (72.4%) occurred in male patients and all cases occurred in the setting of osteoplasty performed for symptomatic FAI. The authors concluded that Indomethacin-based NSAID protocols for HO prophylaxis should be considered after hip arthroscopy in this patient population (Table I).

Beckman *et al*. [36] prospectively reviewed the role of Naproxen prophylaxis after hip arthroscopy. The rate of HO in the control (no prophylaxis) group was 25% (23/92) and in the study group the rate was 5.6% (11/196). The author further identified that patients undergoing acetabuloplasty along with osteochondroplasty were more likely to develop HO. In conclusion, the authors stated that routine NSAID prophylaxis reduces but does not eliminate the incidence of HO in patients undergoing hip arthroscopy (Table I).

### Complications of prophylaxis treatment

Radiation for the prevention of HO carries a potential risk for malignant transformation. Reports of tumor induction by ionizing radiation in low doses are few. Even though one study found no evidence of tumor induction in any patient receiving <3000 cGy [55], other studies reported several cases of neural tumors arising after treatment of tinea capitis with doses of 1000–2000 cGy [56] and radiation-induced bone sarcomas after radiation exposure as low as 1200 cGy [57]. Moreover, doses of 1000–3000 cGy carry a relative risk for oncogenesis 10-fold greater than doses of <1000 cGy [58].

NSAID administration can potentially cause agent dependent adverse effects. Gastrointestinal tract intolerance, platelet inhibition and negative drug interaction with anticoagulation agents such as warfarin are contraindications and adverse effects that must be considered prior to indomethacin use [59]. The use of COX-2 selective inhibitors, especially rofecoxib, has potential adverse cardiovascular complications, particularly in patients with a history of cardiac disease and should be carried out with extra caution in these patients [60].

### Surgical treatment—excision of HO

HO after hip arthroscopy is an asymptomatic incidental finding in most patients [4, 61]. However, it was suggested that revision surgery for ectopic bone removal may be indicated for one in four patients who develop HO [4, 36]. The decision to perform revision surgery is made on an individual basis and is based on the severity of the degree of functional impairment, pain and range of motion restriction after attempts of conservative non-operative rehabilitation. Surgery should be delayed until complete maturation of the HO process, as resection of immature HO leads to an increased complication rate and increased recurrence rate. Classifying the maturity of HO prior to surgical resection of HO foci is paramount and thus the following guidelines have been established. The radiographic appearance of the ossification should be consistent with dense cortical bone, levels of serum alkaline phosphatase should be within the normal range and bone scan findings should return to normal or near normal [62] which usually occurs within 6–12 months.

Bedi *et al*. [4] treated 7 of 29 patients who developed HO post-operatively with revision surgery to excise ectopic bone at a mean duration of 11.6 months after the index procedure. All of the revised hips had ectopic bone located anterior to the joint. The surgical approach for HO removal was arthroscopic for grade I–II HO and open excision of bone with capsulotomy for hips with grade III or IV HO. Beckman [36] reported arthroscopic ectopic bone resection in 9 of 34 patients who developed HO at >12 months post-operatively. This 12-month period was employed to ensure full maturation of the HO and to allow for adequate recovery from the index surgery.

In the case of revision surgery indicated for the removal of symptomatic HO, prophylaxis with either NSAIDS or radiation is mandatory. As previous HO is a significant risk factor for future occurrences appropriate prophylactic measures must be employed. Complications of the surgical removal of HO include hemorrhage, wound-healing issues, cellulitis, infection and possible recurrence of HO.

## SUMMARY

HO is the abnormal formation of mature lamellar bone within extraskeletal soft tissues. HO is one of the most common complications after hip arthroscopy and may impede the functional outcome of surgery by causing pain, impingement and decreased range of motion. The most likely candidate cell responsible for HO is the MSC, which proliferates into pathologic tissue under the stresses of post-operative tissue damage. Risk factors pre-disposing to the development of HO include male gender, previous hip surgery and history of HO among others. The diagnosis of HO is routinely achieved with X-ray radiography although the optimal timing for X-ray diagnosis of HO is yet to be defined. The location of HO lesions can be further evaluated using CT radiography. There are many prophylactic measures currently available to prevent post-surgical HO, however the most commonly employed agents are NSAIDs. Finally, surgical excision of HO is indicated in patients with a severe degree of functional impairment, pain and range of motion restriction after attempts of conservative non-operative rehabilitation. Furthermore, HO lesions must be allowed to fully mature prior to surgical excision.

## CONFLICT OF INTEREST STATEMENT

None declared.
